# Supplementary material for: Identification of photoperiod-induced specific miRNAs in the adrenal glands of Sunite sheep (Ovis aries)
Source: Front Vet Sci. 2022 Jul 22;9:888207. doi: 10.3389/fvets.2022.888207 (PMC9354845; doi:10.3389/fvets.2022.888207)
Supplement: Supplementary file 1 [file Data_Sheet_1.ZIP › Supplementary file/miRDeep2 file.zip!/miRDeep2 file/Know_Structure/SP42A2/oar-mir-329b.pdf]

miRBase precursor : oar-mir-329b
Total read count : 3116
oar-miR-329b-5p read count : 11
oar-miR-329b-3p read count : 3096
remaining reads : 9

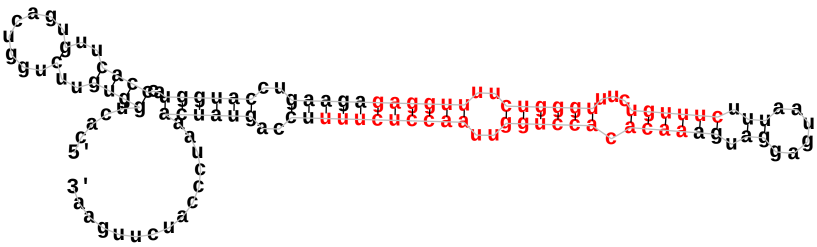

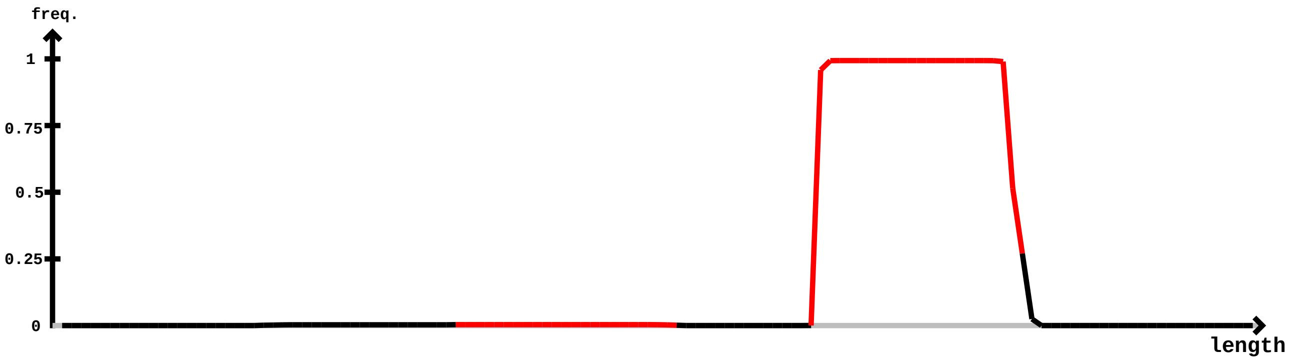


```
                                                                                oar-miR-329b-3p
                                         oar-miR-329b-5p
5'- cacugguguucuggucaguguucacccaugguaccugaagagagguuuucuggguuucuguuucuuuaaugaggaugaaacacaccugguuaaccucuuuuccaguaucaaaucccaucuugaa  -3'    exp
    ....((((..(........)..))))..((((((..(((((((((((..((((((...((((((.((......)).)))))).))))))..)))))))))))..))))))..............  reads   mm
    ....................uucacccaugguaccugaaga...................................................................................  4       0
    .....................ucacccaugguaccugaaga...................................................................................  2       0
    ......................cacccaugguaccugaaga...................................................................................  2       0
    .......................acccaugguaccugaag....................................................................................  1       0
    ........................................agagguuuucuggguuucugu...............................................................  1       0
    ........................................agagguuuucuggguuucuguu..............................................................  1       0
    ........................................agagguuuucuggguuucuguuuc............................................................  1       0
    .........................................gagguuuucuggguuucuguu..............................................................  1       0
    .........................................gagguuuucuggguuucuguuu.............................................................  4       0
    .........................................gagguuuucuggguuucuguuuc............................................................  3       0
    .............................................................................aaacacaccugguuaaccucu..........................  1       0
    ..............................................................................aacacaccugguuaaccu............................  1       0
    ..............................................................................aacacaccugguuaaccuc...........................  10      0
    ..............................................................................aacacaccugguuaaccucu..........................  1466    0
    ..............................................................................aacacaccugguuaaccucuu.........................  758     0
    ..............................................................................aacacaccugguuaaccucuuu........................  734     0
    ..............................................................................aacacaccugguuaaccucuuuu.......................  16      0
    ...............................................................................acacaccugguuaaccuc...........................  1       0
    ...............................................................................acacaccugguuaaccucu..........................  12      0
    ...............................................................................acacaccugguuaaccucuu.........................  5       0
    ...............................................................................acacaccugguuaaccucuuu........................  30      0
    ...............................................................................acacaccugguuaaccucuuuu.......................  61      0
    ................................................................................cacaccugguuaaccucuuu........................  1       0
```
